# Audit of Early Mortality among Patients Admitted to the General Medical Ward at a District Hospital in Botswana

**DOI:** 10.5334/aogh.1354

**Published:** 2019-03-04

**Authors:** Colleen Kershaw, Margaret Williams, Saikiran Kilaru, Rebecca Zash, Kitenge Kalenga, Felly Masole, Roger Shapiro, Tomer Barak

**Affiliations:** 1 Dartmouth-Hitchcock Medical Center, Lebanon, NH, US; 2 Beth Israel Deaconess Medical Center, Boston, MA, US; 3 The Ohio State University Wexner Medical Center, Columbus, OH, US; 4 New York University School of Medicine, NY, US; 5 Harvard T.H. Chan School of Public Health, Boston, MA, US; 6 Botswana-Harvard AIDS Institute Partnership, Gaborone, BW; 7 Scottish Livingstone Hospital, Molepolole, BW; 8 Gaborone Private Hospital, Gaborone, BW

## Abstract

**Background:**

Mortality among adult general medical admissions has been reported to be high across sub-Saharan Africa, yet there is a paucity of literature on causes of general medical inpatient mortality and quality-related factors that may contribute to the high incidence of deaths. Based on a prior study at our hospital as well as our clinical experience, death early in the hospitalization is common among patients admitted to the adult medical wards.

**Objective:**

Quantify early inpatient mortality and identify factors contributing to early in-hospital mortality of medical patients in a resource-limited hospital setting in Botswana.

**Methods:**

Twenty-seven cases of patients who died within 48 hours of admission to the general medical wards at Scottish Livingstone Hospital in Molepolole, Botswana from December 1, 2015–April 25, 2016 were retrospectively reviewed through a modified root cause analysis.

**Findings:**

Early in-hospital mortality was most frequently attributed to septic shock, identified in 20 (74%) of 27 cases. The most common care management problems were delay in administration of antibiotics (15, 56%), inappropriate fluid management (15, 56%), and deficient coordination of care (15, 56%). The most common contributing factors were inadequate provider knowledge and skills in 25 cases (93%), high complexity of presenting condition in 20 (74%), and inadequate communication between team members in 18 (67%).

**Conclusions:**

Poor patient outcomes in low-and middle-income countries like Botswana are often attributed to resource limitations. Our findings suggest that while early in-hospital mortality in such settings is associated with severe presenting conditions like septic shock, primary contributors to lack of better outcomes may be healthcare-provider and system-factors rather than lack of diagnostic and therapeutic resources. Low-cost interventions to improve knowledge, skills and communication through a focus on provider education and process improvement may provide the key to reducing early in-hospital mortality and improving hospitalization outcomes in this setting.

## Background

The significant gains made in Botswana over recent years in the fight against HIV/AIDS have led to a decline in HIV-associated morbidity and mortality [1] and allowed the country’s Ministry of Health and Wellness (MoHW) to shift the focus of national efforts to general health-systems strengthening and the prevention of morbidity and mortality from both communicable and non-communicable diseases [2]. Across Africa, including Botswana, previous efforts to assess health facility performance tended to rely more on the quantity rather than the quality of care provided [3]. However, in recent years, Botswana’s MoHW has refocused efforts towards quality improvement, and has introduced clinical audits as a primary process for assessing compliance with national health quality standards and promoting quality in healthcare [4]. The reduction of mortality among patients admitted to public sector hospitals in Botswana has been designated as an important goal of such quality improvement efforts.

Mortality among adult general medical admissions has been reported to be high across sub-Saharan Africa [5,6,7], and yet there is a paucity of literature on causes of general medical inpatient mortality and quality-related factors that may contribute to the high incidence of deaths. Based on a prior study at our hospital [8], as well as our clinical experience, death early in the hospitalization is common among patients admitted to the adult medical wards.

We postulated that early in-patient mortality is associated, at least in part, with systems and management problems that may be modifiable and amenable to quality improvement interventions. Using a root cause analysis framework as a form of clinical audit, we aimed to describe the patient and disease characteristics, as well as the underlying systems and management problems, associated with death within 48 hours of admission to the adult male and female medical wards at Scottish Livingstone Hospital (SLH), in Molepolole, Botswana. The results of this study were intended to guide development of quality improvement initiatives aimed at decreasing early mortality among inpatients at SLH.

## Methods

### Study site

The study was conducted at Scottish Livingstone Hospital (SLH), a district hospital in Molepolole, the largest urban village in Botswana, and the main referral center for the Kweneng East district, which has population of 256,752 [9]. District hospitals in Botswana occupy the middle of a hierarchy topped by tertiary referral centers, followed by district hospitals, and finally primary hospitals, clinics, and health outposts at the most local level. SLH has 350 inpatient beds and includes wards for pediatrics, obstetrics and gynecology, surgery, ophthalmology, adult medicine and a 6-bed intensive care unit. Available laboratory resources include complete blood counts, basic chemistry panels, liver function tests, coagulation panels, CD4 counts, HIV-quantitative PCR, blood gases and point of care glucose testing. Microbiological testing is possible but preparation and interpretation of microscopic slides and culture data can be inconsistent. Stock-outs of reagents and machine breakdowns frequently interfere with regular availability of laboratory test results. Available imaging modalities include plain X-rays, which are interpreted by the ordering physician and ultrasound, which is performed and interpreted by ultrasound technicians. CT scans may be performed at a tertiary referral hospital but involve transport of the patient 60 kilometers each direction to and from the hospital. While medication stock-outs are not infrequent, life-saving medication such as antibiotics, intravenous fluids, steroids, antiepileptics, vasopressors and anti-arrhythmic medications are usually available. Blood products such as packed red blood cells and fresh frozen plasma are supplied by the hospital’s blood bank, but availability varies depending on national supply.

There are approximately 100 admissions per month to the male and female medical wards. Patients are usually admitted to the wards by a doctor in the Accident and Emergency Department (A&E) where coverage by a medical officer (i.e. a licensed medical doctor without specialization training) is available 24 hours, 7 days a week. During weekday working hours, an internal medicine specialist and a team consisting of a medical officer and rotating medical interns in training oversee each ward. During off-hours, weekends, and holidays, the admitting team consists of one medical officer and one medical intern in the A&E. Inpatients are managed by a single team consisting of one medical officer and one medical intern who provide coverage for all of the hospital’s inpatient wards.

### Study investigators

This study was performed as an internal audit at SLH in collaboration with the hospital’s management and quality improvement team. The case-reviewing investigators were US-trained internal medicine specialists who were deployed at the hospital for six-month-long clinical rotations as part of an international capacity building collaboration under the Botswana-Harvard AIDS Institute Partnership.

### Case enrollment

The medical records of all patients discharged from the male and female medical wards were reviewed daily from December 1, 2015 through April 25, 2016. Cases of “early death” were defined as patient death within 48 hours of admission to the medical wards. Death during this timeframe was considered to be an adverse event for root cause analysis purposes. Time of admission was defined as triage time in the A&E and time of death was the time on the certification note by a physician, or, if missing, the time of death noted in nursing notes.

### Case review procedures

Detailed chart review and root cause analysis of each death was performed by two of three study investigators (CK, SK, MW) who worked as attending physicians/internal medicine specialists on the medical wards at SLH. Two investigators reviewed each case. Discordant findings were reconciled through discussion between the investigators.

### Root cause analysis

Root cause analysis was standardized by use of a study template, which was adapted from the Vincent Framework [10] to fit the local healthcare context in Botswana. The study template required case-reviewing investigators to summarize patient demographics, presenting symptoms, clinical findings, hospital course, and most probable causes of death. Presenting signs and symptoms were assigned by the reviewers if either the A&E or admitting physician’s notes specifically named an element of presentation or if review of the record showed that the patient met clinical parameters defining each sign or symptom. Respiratory distress was identified by the reviewers when recorded vital signs included a respiratory rate greater than 20 breaths per minute, pulse oximetry reading below 92%, or if comments recorded by the A&E or admitting physician suggested respiratory difficulty. Tachycardia was defined as a recorded heart rate equal to or greater than 100 beats per minute, and hypotension was defined as a recorded systolic blood pressure less than 90 mmHg. Altered mental status was identified by the reviewers if the A&E triage sheet recorded the patient’s mental status as being non-alert and “responsive to verbal stimulus”, non-alert and “responsive to pain” or “non-responsive” or if the A&E or admitting physician’s notes indicated altered mentation, “confusion” or a Glasgow coma score of less than 15. Cause of death determination in this study reflects the reviewing investigators’ best judgment based on retrospective review of the patient’s medical record and root cause analysis.

Investigators were also required to identify “care management problems” which occurred in the course of each patient’s hospitalization. These were defined as errors or missed opportunities. No limit was set to the number of problems that could be identified. Specific care management problems were subsequently classified into categories to facilitate the identification and reporting of patterns. For each care management problem, at least one “contributory factor” had to be identified. Contributory factors were standardized by the Vincent Framework and defined as the conditions in which errors may occur. Finally, the case-reviewing investigator was required to estimate the likelihood that death could have been prevented, selecting the degree of preventability from a continuum of five choices ranging from “very unlikely” to “very likely.”

## Results

Between December 1, 2015 and April 25, 2016 there were 514 admissions to the adult general medical wards at SLH. Of those admitted, 92 (18%) patients died. Of deaths, 31 (34%) occurred within 48 hours of admission and were initially enrolled as cases in our study. Four (13%) cases were excluded due to unavailability of the complete patient records at the time the review was performed. The study thus includes root cause analyses and results from 27 (87%) of the 31 early deaths occurring during the study period.

Patients in whom early death occurred included 13 males (6% of all admissions to the male medical ward) and 14 females (5% of all admissions to the female medical ward). Among those who died within 48 hours of admission, the median age was 68 years (range 27–96), HIV status was positive among 10 (37%), negative among 11 (41%) and unknown in 6 (22%). Differences were observed by day of admission, with the highest number of early mortality cases admitted on Monday (30%) and the fewest on Thursday (4%) with otherwise relatively even distribution. Eight patients (30%) died following admission but before being reviewed by a doctor on the medical ward (Table 1).

**Table 1:** Patient and Admission Characteristics.

PATIENT AND ADMISSION CHARACTERISTICS	Number of cases (%) (N = 27)

Female	14 (52)
Male	13 (48)
Median age	68
HIV status	
Positive	10 (37)
Negative	11 (41)
Unknown	6 (22)
Day of admission	
Monday	8 (30)
Tuesday	4 (15)
Wednesday	4 (15)
Thursday	1 (4)
Friday	4 (15)
Saturday	2 (7)
Sunday	4 (15)
Seen by ward doctor prior to death	
Yes	19 (70)
No	8 (30)

### Initial presentation

Respiratory distress was the most common presenting sign, present in 20 (74%) of the 27 cases. This was followed by altered mental status, present in 17 (63%); hypotension, present in 11 (41%); and tachycardia, present in 9 (33%). Presenting symptoms, signs, and abnormal initial investigation findings are presented in Table 2.

**Table 2:** Findings at Presentation.

Presenting Sign or Symptom	Number of cases (%) (N = 27)

Respiratory distress	20 (74)
Altered mental status	17 (63)
Hypotension	11 (41)
Tachycardia	9 (33)
Vomiting and/or diarrhea	5 (19)
Fever	5 (19)
Chest pain	4 (15)
New onset hemiparesis	2 (7)
Collapse	1 (4)
Hypertension	1 (4)
Jaundice	1 (4)
Seizure	1 (4)
Night sweats	1 (4)
Dizziness	1 (4)
Hypothermia	1 (4)
Headache	1 (4)
**Abnormal findings on initial investigations**	
Renal failure	5 (19)
Hypokalemia	3 (11)
Hypoglycemia	3 (11)
Hyperkalemia	3 (11)
Myocardial ischemia on ECG	1 (4)
Hyperglycemia	1 (4)
Severe anemia	1 (4)
Hyponatremia	1 (4)

### Causes of death

Up to two probable causes of death were identified for each patient. The most common probable cause of death was septic shock, identified in 20 (74%) of 27 cases. The majority of the sepsis cases studied were thought to be due to a pulmonary source: either pulmonary tuberculosis (TB) or a non-TB pulmonary pathogen. Presumed or known tuberculosis-driven sepsis was thought to be a major factor leading to death in 10 cases (37%). A non-tuberculosis pulmonary source of sepsis was considered the likely cause of death in an additional six cases (22%), and in three of the 20 sepsis cases no obvious source was identified. Other less common probable causes of death are summarized in Table 3.

**Table 3:** Probable Causes of Death.

Probable Cause of Death	Number of cases (%) (N = 27)

Septic shock:	10 (37)
Pulmonary TB (presumed or known)	
Septic shock:	6 (22)
Pulmonary, presumed non-TB pathogen	
Septic shock:	1 (4)
Extrapulmonary TB (presumed)	
Septic shock:	3 (11)
Unclear source	
Pulmonary edema	4 (15)
Cardiac shock	2 (7)
Aspiration pneumonia	2 (7)
Meningoencephalitis	2 (7)
Lymphoma	2 (7)
Hyperkalemia/renal failure	2 (7)
Diabetic ketoacidosis	1 (4)
Liver failure	1 (4)

### Care management problems

Root cause analysis revealed multiple care management problems in each of the cases reviewed, with a mean of 4.7 (range of 2–9) problems identified per early death case. The most common care management problems identified were delay in the administration of antibiotics in 15 (56%) patients, inappropriate fluid management in 15 (56%) patients, and deficient coordination of care in 15 patients (56%). Problems with fluid and antibiotic management were identified through review of orders and medication administration records in the patients’ charts. Fluids or antibiotics ordered or administered more than one hour after admission were considered to be delayed. Delay in administration of antibiotics was due to failure of the managing doctor to order antibiotics in some cases. In other cases, no clear reason for the delay could be identified, as antibiotics were ordered but never administered, or administered hours later. Inappropriate fluid management consisted in most cases of inadequate fluid resuscitation for septic shock, usually due to either failure to order fluids, or failure to administer ordered fluids, for which no clear reason was documented. In one case, inappropriate fluid management consisted of administration of excess fluids.

Deficiency in coordination of care was identified when timely and appropriate management was judged by the case reviewers to have been delayed or prevented due to the lack or failure of communication between healthcare providers. This included incompletely or incorrectly executed orders, lack of handoff between the admitting doctors in the A&E and the doctors covering the wards, failure of medical officers to seek advice from the on-call specialist, and cases of failure of doctor-nurse and nurse-nurse communications that were evident in the medical record.

Importantly, the three most common care management problems in the study were also the most common problems identified among the 20 cases in which sepsis was the presumed cause of death [delay in antibiotics in 15 of the 20 presumed sepsis cases (75%), inappropriate fluid management in 13 (65%), and deficient care coordination in 12 (60%)]. An overview of all care management problems identified among the 27 early mortality cases is shown in Table 4.

**Table 4:** Care Management Problems.

Care Management Problems	Number of cases (%) N = 27

Delay in administration of antibiotics	15 (56%)
Inappropriate fluid management	15 (56%)
Deficient coordination of care	15 (56%)
Inadequate management of hypoxia	13 (48%)
Lack of infectious workup	11 (41%)
Imaging studies not done	11 (41%)
Unavailability of laboratory test results	11 (41%)
Inadequate follow-up of laboratory test results	10 (37%)
Inadequate patient monitoring	9 (33%)
Inadequate management of electrolytes	6 (22%)
Inappropriate medication management	4 (15%)
Delay in MD evaluation	3 (11%)
Arrhythmia not addressed	3 (11%)
Lack of access to intensive care	3 (11%)
Inadequate management of heart failure	2 (7%)
Lack of recognition of cardiac ischemia	2 (7%)
Delay in blood transfusion	2 (7%)
Delay in lumbar puncture	2 (7%)
Unavailability of necessary medication	1 (4%)

### Contributory factors related to care management problems

**Figure 1:**
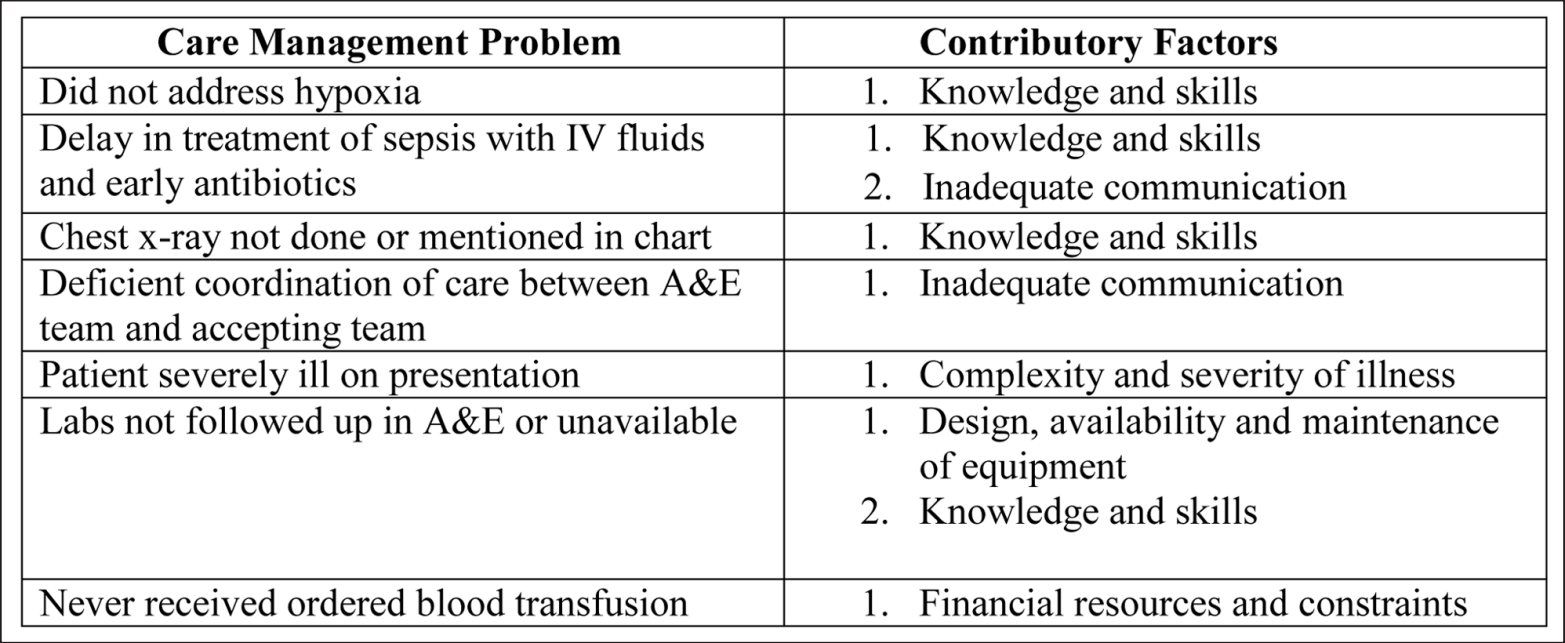
Sample Data Collection Study Template.

In each of the cases reviewed, multiple factors were identified as contributing to care management problems, with a mean of six contributing factors (range 3 to 9) per early death case and a range of 1 to 3 factors contributing to each care management problem. Figure 1 provides an example of the relevant portion of the data collection tool for one case of early death in which the probable cause of death was assessed as sepsis from a pulmonary source.

The most common contributing factor, identified in 25 (93%) of the cases reviewed, was inadequate provider knowledge and skills, as assessed by the reviewers’ chart examination. Inadequate provider knowledge and skills was selected as a contributing factor to patient early death if the provider did not adequately identify the emergent medical condition as determined by reviewers’ assessment of the available information of the patient’s presentation, or failed to follow appropriate diagnostic and treatment protocols based on the likely diagnosis. For example, if a patient presented with a clinical picture suggestive of sepsis but sepsis was not recorded as considered in the differential diagnosis or if sepsis was considered but appropriate diagnostic and therapeutic steps (e.g. cultures, fluids, antibiotics) were not instituted, the reviewers determined that the provider had failed to identify the emergent presenting condition or failed to follow the standard of care based on the likely diagnosis. Among the 20 sepsis cases, which accounted for the majority of deaths, deficiency in knowledge and skills was cited as a contributing factor in 19 (95%). This deficiency was identified in all of the sepsis death cases in which delay in antibiotics or inappropriate fluid management were identified as care management problems.

The second most common contributing factor was high complexity of the presenting condition in 20 (74%), and inadequate verbal and written communication between team members in 18 (67%). A summary of identified contributing factors is presented in Table 5.

**Table 5:** Contributory Factors.

Contributory factor	Number of cases (%) N = 27

Knowledge and skills	25 (93%)
Complexity of condition	20 (74%)
Verbal and written communication	18 (67%)
Maintenance and availability of equipment	17 (63%)
Availability and use of protocols	16 (59%)
Availability of test results	15 (56%)
Staffing levels	13 (48%)
Financial resources and constraints	11 (41%)
Failure of team leadership	8 (30%)
Policy standards and goals	6 (22%)
Patterns in workload/shift	3 (11%)
Administrative and managerial support	3 (11%)
Safety culture and priorities	2 (7%)
Willingness to seek help	1 (4%)
Patient personality and social factors	1 (4%)

### Likelihood that death could have been prevented

Among the 27 cases of early death reviewed in the study, one case was rated as “very likely” to have been preventable. This was a case of diabetic ketoacidosis in which the admitting team failed to correctly identify the presenting problem and subsequent management was compounded both in the A&E and on the inpatient ward by inappropriate fluid management and inadequate monitoring. Four deaths (15%) were rated “likely”, 11 (41%) were rated “uncertain,” 5 (19%) were rated “unlikely”, and 6 (22%) were considered “very unlikely” to have been preventable. In the 20 cases attributed to sepsis, zero were rated “very likely” preventable. One (5%) was rated likely, 9 (45%) were rated uncertain, 4 (20%) were rated “unlikely” and 4 (20%) were rated “very unlikely” preventable.

## Discussion

We reviewed deaths within 48 hours of hospital admission to the adult medical wards, and identified care management problems that may have contributed to these deaths at a district hospital in Botswana. Early mortality frequently involved high complexity and severity of illness, and septic shock was identified as the probable cause of death in most cases. In this study, which aimed to identify opportunities for quality improvement interventions in a relatively low resource setting, the most notable finding was that factors contributing to care management problems more often involved limitations in education and training of personnel and inadequate communication between healthcare providers, rather than constraints on physical resources such as availability of medications or equipment for diagnosis and therapy.

While the study was not designed to identify the initial presentations that have the highest odds ratios for early mortality, the most common presenting signs and symptoms were likely reflective of shock states: respiratory distress, altered mental status, hypotension, and tachycardia. Most patients had more than one of these signs and symptoms, and most cases reviewed in the study were suspected to reflect septic shock. Intravenous fluids and antibiotics are the cornerstone of sepsis management; [11] however, despite their ready availability at SLH, the most commonly identified care management problems were delay in delivery of antibiotics and inappropriate or inadequate fluid management. Delays in administration of fluids and antibiotics occurred for a myriad of reasons; in cases where antibiotics and fluids were never ordered, or there was a significant delay in ordering, review of the medical documentation suggested that clinicians often did not recognize sepsis as a probable cause of illness and, in cases where sepsis was considered, may not have appreciated the urgent need for intervention. This was thought to represent a deficiency in provider knowledge and skills, identified as one of the most common contributory factors to early deaths.

In some cases fluids and/or antibiotics were ordered but not administered or administered with significant delays. Some delays seemed to have been caused by miscommunication or confusion regarding immediate (i.e. “STAT”) dosing versus routine timed administration. In other cases lack of communication between nurses and prescribers resulted in failure to alert ordering providers when a specific antibiotic or fluid were not available and to identify alternatives for out-of-stock medications. The recognition, workup, and immediate management of sepsis and septic shock according to established protocols and the introduction of appropriate documentation and closed-loop communication protocols should thus be a focus for education and quality improvement interventions in our setting.

Deficient coordination of care between providers was also identified among the most common care management problems, and inadequate communication as described above was a common contributory factor. Alarmingly, in thirty percent of reviewed early death cases, patients died following initial review by a doctor in the A&E and admission to the medical ward but before being seen by the accepting doctor on the ward. In most of these cases no documentation existed of any handoff communication between A&E staff and ward staff, whether physician to physician, physician to nurse, or nurse to nurse. This likely reflected both a failure to recognize critically ill patients who require close monitoring and follow-up and the absence of established hospital protocols for patient handoffs and transfers of care between the A&E department and the inpatient wards. The lack of readily available communication equipment is an additional barrier that may need to be addressed if such protocols are to be successfully implemented in the future.

Shortage of staffing and high patient-to-provider ratios are pervasive issues in Botswana, as in many other public healthcare systems in resource-limited settings and contribute to many of the care management problems identified in this study. This is especially problematic during weekend and off hours when only two medical officers (each assisted by an intern in training) are available to provide coverage for the entire hospital, one stationed in the A&E department and the other covering all inpatient wards including medical, surgical, obstetrics & gynecology and pediatrics. During the period of this study, routine practice at SLH was that a patient would not be seen by a doctor on the ward until Monday if admitted between Friday evening and Monday morning unless called by a nurse regarding an emergent clinical situation. We therefore expected that patients admitted over the weekend would be at higher risk of early inpatient death. In this context, while this study was not designed or powered to detect differences in rates of mortality by day of presentation, the finding that more cases of early in-hospital mortality occurred among patients presenting to the A&E on Mondays was nevertheless unexpected, as we hypothesized a higher percentage of deaths would occur among weekend admissions. One possible explanation is that medical staff returning to the wards on Monday mornings are overwhelmed by the clinical needs of patients who were admitted over the weekend, thus preventing them from addressing new weekday admissions in a timely manner.

## Limitations

A number of important limitations must be considered in interpreting the results of the present study. The study included a small number of cases over a short period of time. It was conducted through a detailed root cause analysis aimed at identifying care management problems that may have contributed to early in-hospital mortality, but was not designed to prove a causative relationship between care management problems and poor outcomes, nor to identify patient risk factors for early in-hospital mortality. The study was conducted as an internal hospital audit and performed by staff clinicians who, in some cases, were involved in the care of patients under review, which may have biased the findings. Future audits in this area would benefit from an independent multidisciplinary team of reviewers. Additionally, limitations in diagnostics and post-mortem investigations restricted our ability to determine a definitive cause of death for most patients. This significantly limited our ability to weigh the relative contribution of identified care management problems to each outcome and to estimate the likelihood that death could have been prevented.

Further, we rarely had access to outpatient medical records and were not able to assess the impact of pre-hospital care, which may have contributed to advanced disease states at presentation, especially in our setting. For example, these could have included patients’ care seeking behavior, cultural attitudes towards traditional healing practices and modern biomedicine, delays in appropriate diagnosis and management in community level and referring facilities, distance from the hospital, and availability of transport. A significant reduction in early in-hospital mortality may require interventions at the community and referring facility level in addition to those aimed at improving care management at the district hospital.

Importantly, some of the limitations we faced in the process of conducting the current study highlight challenges in quality improvement work likely to be common in many other resource-limited settings. While we found the Vincent Framework [10] to be a helpful guide, several components of root cause analysis were limited in comparison to similar audits conducted in high resource settings. Lack of standardized clinical documentation was a significant barrier to a reliable retrospective recreation of patients’ hospital courses; issues not specifically documented in the patient’s chart, such as equipment malfunction and inadequate staffing, are missing from our analysis. Poor quality and availability of medical records have been reported in other retrospective reviews of healthcare quality in developing countries [12] and previous clinical audits conducted in Botswana [13].

Root cause analyses typically include interviews with staff members that were involved in the cases undergoing review. This was not done in the course of the current study because the work presented here represents a relatively new approach for our institution where the protected, non-judgmental environment required to foster open discussion is only beginning to be established. We hope this work will contribute to the development of an organizational culture geared toward quality-improvement in which staff members feel comfortable discussing and learning from shortcomings and poor outcomes.

## Conclusions

Our findings suggest that septic shock is the most common cause of early in-hospital mortality at our institution, and that despite available diagnostic and therapeutic resources, recognition of septic shock and interventions for basic management were inadequate, as was coordination of care between providers. Quality improvement interventions focused on the identification and early management of sepsis and septic shock and improvement of inter-provider communication may be effective in reducing early mortality among adults admitted to the medical wards in our hospital. While physical resource limitation is often seen as the major barrier to improving health outcomes in low- and middle-income countries, our findings suggest that improving the utilization of existing resources through a focus on provider training and internal process improvement may improve outcomes even in the absence of costly investment in health infrastructure and material resources. As a next step at our hospital, we have embarked on the development and implementation of a sepsis recognition and management tool as well as the introduction of a low-cost hand-held radio communication system to improve care coordination between providers [14]. We plan to report on the impact of these interventions in the future.

In conclusion, our study provides a novel addition to the literature, as little has been published about the factors contributing to high inpatient mortality in similar resource-limited settings in sub-Saharan Africa. While this report highlights various challenges to conducting clinical audits and root cause analyses in resource-limited settings, it also points to the opportunities inherent in quality improvement efforts in this healthcare context. Notwithstanding the challenges, as healthcare professionals, we are obligated to continually improve the quality of the care that we provide. This study represents a starting point for this process in a resource-limited district hospital setting.
